# Pharmacists’ Confidence in Managing Patients with Inflammatory Bowel Disease

**DOI:** 10.3390/pharmacy8020068

**Published:** 2020-04-17

**Authors:** Sharmila S. Prasad, Simon Keely, Nicholas J. Talley, Therése Kairuz, Marjorie M. Walker

**Affiliations:** 1 Faculty of Health and Medicine, School of Biomedical Science and Pharmacy, University of Newcastle, Callaghan, New South Wales 2308, Australia; simon.keely@newcastle.edu.au (S.K.); therese.kairuz@newcastle.edu.au (T.K.); 2 Priority Research Centre, Digestive Health and Neurogastroenterology, University of Newcastle, New Lambton Heights, New South Wales 2308, Australia; nicholas.talley@newcastle.edu.au (N.J.T.); marjorie.walker@newcastle.edu.au (M.M.W.); 3 Faculty of Health and Medicine, School of Medicine and Public Health, University of Newcastle, New Lambton Heights, New South Wales 2308, Australia

**Keywords:** cross-sectional survey, continuing education, pharmacy practice, inflammatory bowel disease, pharmacist, professional

## Abstract

**Background and aim**: Managing patients with a chronic condition such as inflammatory bowel disease (IBD), requires a multidiscipline approach. The pharmacist might be the first point of contact for patients with initial symptoms or relapsing flares, yet there is no available literature on the role of pharmacists in IBD management. We conducted a survey to explore pharmacists’ confidence in and potential barriers to managing IBD and assess the impact of IBD education on their confidence in IBD management. **Methods**: Surveys assessing confidence levels in managing IBD, additional learning opportunities about IBD and barriers to their learning of IBD management were provided to pharmacists for completion before and after attending an IBD-specific education session at a national conference. **Results**: Of the 195 attendees, 125 participants completed the survey (64%). Most respondents reported a low to mid-range level of confidence with managing IBD. Specifically, they were only slightly confident in decision making on patient care, addressing patient needs and providing additional support for IBD patients; and somewhat confident with understanding, management and providing relevant information on IBD. Whist the education session improved pharmacists perceived level of confidence, most respondents indicated a need to learn more about IBD. Areas of additional learning included science, drug therapy, treatments (includes non-pharmacological options as well) and guidelines. A majority of pharmacists identified time constraints as a key barrier to learning. **Conclusion**: Pharmacists lack sufficient confidence about managing inflammatory bowel disease. These data indicate support within the pharmacy profession to play a more active role in the management of IBD.

## 1. Introduction

Inflammatory bowel disease (IBD) is a term used to describe a group of chronic intestinal inflammatory diseases and the two most common are ulcerative colitis (UC) and Crohn’s disease (CD) [1,2]. IBD is associated with a range of debilitating symptoms, such as severe abdominal pains, urgent and frequent diarrhoea, faecal incontinence, anaemia, anxiety and depression that affect physical and mental health [3,4,5]. It is reported that IBD sufferers overall have a poorer quality of life, especially during the active disease state [5,6,7]. Specifically, individuals with IBD can have a range of issues that impact daily activities, for example, limited participation in social activities and frequent absence from work/school. Fears specifically centre on the need to be close to toilet facilities and the consequences of bowel incontinence [4,8,9].

IBD is a complex and life-long condition that involves adaptation to treatment regimens and fluctuating symptoms [1]. IBD management, however, has advanced significantly in recent decades and the treatments are becoming more diverse with the availability of newer agents [10]. Patients with IBD have distinctive therapeutic, behavioural and preventive care requirement [11,12,13]. Managing IBD patients involves treatments aimed at induction and maintenance of remission while improving quality of life [1,2,10]; and the gastroenterologist is generally the principal care provider in this cohort [14]. Patients are often provided with information but their perceptions of the risk associated with IBD-related treatment vary widely [8]. 

Many challenges still exist in obtaining optimal care for patients with IBD and are encountered on a daily basis in all healthcare settings (primary, secondary and tertiary care); healthcare professionals therefore have the responsibility to effectively communicate treatment regimens which have the potential to influence disease outcomes. There are no defined guidelines for pharmacists in the management of IBD [7,15,16,17,18]. Several studies show components of pharmacists’ management of IBD patients involving dug monitoring, medication counselling and follow up appointments allowing assessment of both clinical response and safety monitoring in secondary and tertiary care settings [19,20,21]. 

Despite multiple studies documenting perceptions of healthcare professionals regarding IBD management, the majority focus on specialist medical or tertiary care [22,23,24,25]. There are no data concerning pharmacists’ knowledge of and exposure to IBD. Pharmacists’ perceptions of barriers to the care and management of patients with IBD have also not been previously described. This survey was therefore aimed at identifying the level of confidence pharmacists have in care and management of patients with IBD. This study was conducted as a face-to-face survey that was distributed to delegate attendees before an educational session on IBD at a national conference. Our secondary aim with this survey was to identify any perceived barriers to the conference attendees’ exposure to IBD care or management.

## 2. Materials and Methods

### 2.1. Survey Development

The IBD Confidence Survey was a cross sectional survey developed to obtain pharmacists’ perceptions of the primary care management of IBD and of any barriers to their learning and to gather additional information for future opportunities to enhance IBD management.

The survey was piloted for face validity among a small sample size of 10 young adults and five academic pharmacists and to determine whether the survey adequately elicited the confidence level of pharmacists in IBD management. The survey was revised accordingly prior to ethics approval and distributed at the PSA19 national conference held in New South Wales, Australia. The revisions included, modifying the Likert scale from a 10-scale to a 5-point scale, simplifying statements, limiting the number of free-text questions and shortening the length of the survey for completion within a reasonable time-frame. The final version of the survey comprised two sections (Supplementary Information); the first section included five statements on understanding IBD, and management of IBD, provision of relevant information about IBD, making decisions about patient care and providing additional support for IBD patients while addressing patient needs; which was to be completed before the education session. The second section consisted of seven statements and three additional questions (two free text questions) that focused on barriers to learning, future opportunities for information on IBD management and attending future educational sessions on IBD (Table 1).

Although the two sections were not identical, both measured pharmacists’ confidence in the following: understanding IBD and its management, providing relevant information, decision-making, additional support and addressing the needs of patients. The second section was designed to focus and demonstrate specific representation of some of the statements being assessed prior to the education session. For example, understanding and management looked at the level of confidence of pharmacist’s ability to identify signs and symptoms of IBD, distinguish between IBD and other gastrointestinal disease and when to refer patients. The statement about making decisions with IBD patients about their care in the first section was assessed as being able to review/monitor treatments and provide tailored guidance on adherence/compliance. In addition, the statement on additional support was measured as being confident in accessing relevant resources on IBD (Table 2).

### 2.2. Data Collection

The survey was distributed to delegates attending an IBD educational session at the PSA19 Conference, Sydney on 27 July 2019. All attendees at the IBD session were provided with a double-sided paper survey containing separate questionnaires for before and after the session. Participants were instructed to complete the relevant surveys before and after the session. The surveys were collected as they left the session and the room was checked for any surveys left behind. Due to time constraints during and between educational sessions, no demographic details were collected as part of this study.

### 2.3. Data Analysis

To assess pharmacists’ perceptions, each question was answered on a Likert scale from 1 to 5 to reflect their level of confidence (1 = not confident; 5 = entirely confident). Manual data were recorded for analysis using Microsoft Excel. The majority of analyses were descriptive, where frequencies (%) were used to describe the data. Free text responses were coded, and similar codes were grouped thematically using a qualitative approach described by Braun and Clarke; this included familiarization with the data, coding, generating themes, reviewing themes, defining and naming themes and writing up the data [26]. 

### 2.4. Ethics and Competing Interests

Whilst the IBD Confidence survey had approval from the Pharmaceutical Society of Australia (PSA), it was an independent survey that was not sponsored or endorsed by the sponsors of the session or the PSA. The study had ethical approval from the Research Ethics Committee at Hunter New England Health [2019/ETH00167] and the University of Newcastle Human Research Ethics Committee (HREC) [H-2019-0201].

## 3. Results

### 3.1. Study Sample

Overall, the number of delegates attending the IBD education session was 200, 195 of whom received the survey (5 late arrivals did not receive the survey). Of these, 46 attendees (24%) did not complete the survey and 24 (12%) returned unanswered surveys. The incomplete and unanswered surveys were removed from the analysis. The final analysis was therefore based on 125 completed surveys. This represented an overall response rate of 64%. 

### 3.2. Pharmacist Confidence

**Figure 1:**
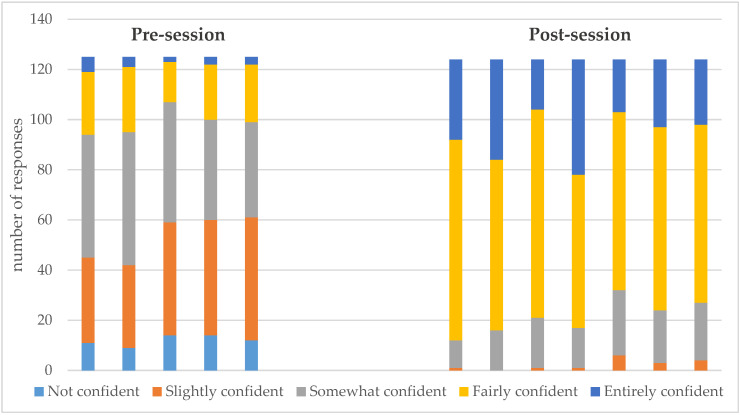
Overall confidence level of pharmacists pre- and post-inflammatory bowel disease (IBD) education session.

**Figure 2:**
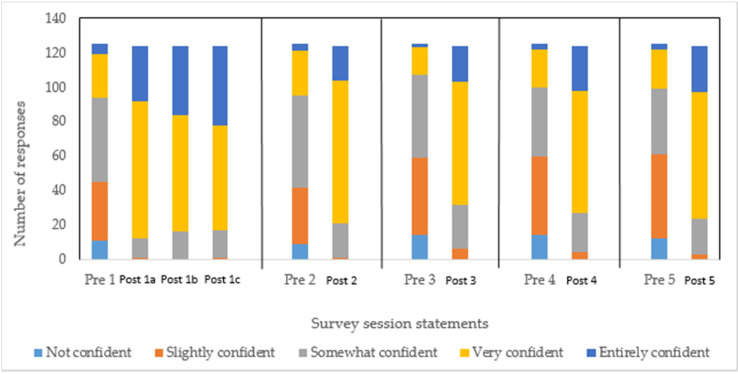
Confidence level of pharmacists for each statement in the pre-session (pre) compared to a parallel post-session statement (post). Pre-statement 1 = understanding IBD and management; post 1a = identifying signs and symptoms of IBD, post 1b = distinguishing IBD from other gastrointestinal diseases, post 1c = recognising when referral is required. Pre- and post-statement 2 = provide relevant information. Pre- and post-statement 3 = decision-making. Pre- and post-statement 4 = additional support. Pre- and post-statement 5 = address needs of patients.

The survey focused on 5 key aspects of IBD, namely, understanding IBD and its management, providing relevant information, decision-making, additional support and addressing the needs of patients. Overall, prior to the education session, the majority of pharmacists reported being slightly/somewhat confident about their understanding of the topic of IBD, as shown in Figure 1. Ten percent of the respondents indicated they were not at all confident about any of these five IBD concepts. Thirty six percent of the respondents felt slightly and somewhat confident, 21% were fairly confident and a very small percentage (3%) of respondents felt entirely confident. Specifically, pharmacists’ level of confidence did not change when they were asked about addressing patient needs, providing additional support for IBD patients and decision making on patient care, as shown in Figure 2. Furthermore, pharmacists were not very confident about providing relevant information on IBD and on management of IBD; 7% reported they did not feel confident, 26% were slightly confident, 43% were somewhat confident and 21% were fairly confident, however, only 3% were entirely confident.

The two additional statements in the post-session survey measured specific aspects in pharmacists’ understanding of IBD, such as being able to identify signs and symptoms of IBD, to distinguish IBD from other gastrointestinal diseases and being able to refer patients, when required. The survey showed a marked improvement in pharmacists’ perception of their level of confidence as a result of the education session (Figure 2). Whilst approximately 58% of the respondents claimed to be now fairly confident and 24% felt entirely confident, there were nevertheless still 15% of pharmacists who felt only somewhat confident. Interestingly, even after the education session, a very small percentage (3%) of pharmacists did not feel confident at all (Figure 1). When considering the confidence levels of pharmacists in addressing patients’ needs, in being able to access relevant information on IBD and in providing tailored information, 58% of pharmacists were fairly confident. However, the level of confidence among the remaining respondents was almost the same between those feeling somewhat confident (22%) and those feeling entirely confident (20%).

### 3.3. Educational Opportunities

Forty-one percent of respondents completed the free text question on further education regarding IBD. The responses were categorised into the following six themes: treatments, IBD management, guidelines, the science of IBD, healthcare professionals and IBD awareness. Approximately one fifth (22%) of the responses related to more than one topic concerning pharmacists’ learning opportunities for IBD, namely, treatments, IBD management and guidelines. The key focus for most pharmacists was to learn more about treatments (36%), the basic science of IBD (21%), IBD management (17%) and guidelines (14%), all being highly relevant topics in the current management of patients with IBD. A small percentage of respondents were also interested about IBD awareness (7%) and the role of healthcare professionals (5%) in IBD management.

Concerning treatments, the focus (16%) was on pharmacists’ willingness to learn more about the difference between each treatment, treatment options available, the administration of therapy (1%) and the mode of action of pharmacological therapies (5%). There was considerable interest in learning more about newer agents, novel or emerging treatments (10%), diet (5%), probiotics (4%), complementary medicines (4%), biologics (4%) and the role of faecal transplants (1%). Other topics of interest were IBD-management-related lifestyle modifications (4%), non-pharmacological approaches (4%), compliance/adherence to therapy (4%) and information on mental health (1%) and support groups for IBD patients (1%). Guidelines were also important for pharmacists; they would like advice and guidance about the stepwise approach to therapy (4%), referral pathways (2%) and therapeutic decision-making about IBD care (2%). The science of IBD was equally important to pharmacists for they wanted to learn more about the aetiology (4%), immunological factors (4%), prevalence/incidence (4%), risk factors (4%), genetics (2%), gut flora (2%), biological markers (2%) and extra-intestinal complications associated with IBD (2%). Although it was only a small interest from some pharmacists, they also wanted to know more about the role of pharmacists in IBD (1%) and about collaborative/integrative management (1%) as well as being made more aware of research into the prevention of IBD (2%).

### 3.4. Barriers to Learning

Only a quarter (25%) of the respondents answered the free text question on barriers to learning. The responses could be divided into the following six themes: time, information, distance, cost, clinicians and practice. Over half (55%) of the pharmacists found that time was a barrier to their learning and approximately a quarter (23%) of them considered information to be a barrier. Other pharmacists found that distance (10%), cost (3%) associated with updating knowledge, access to clinicians (3%) and practice (6%) were barriers to their learning.

Time included having long hours of work and not having enough available time during working hours to allow for additional learning. Pharmacists reported that access to information was limited at their place of work, and not being able to find or to know about good resources on IBD management being potential barriers to their learning. Some pharmacists felt the information delivered during the education session was ‘basic’ and not clinically relevant to their needs. Practice-related pharmacists with a non-medical speciality or impending retirement were also seen as possible barriers to greater awareness of IBD developments.

Pharmacists also found that distance was a factor affecting their ability to update their learning; this was especially true for those working in rural or remote areas and thus having restricted access to key education sessions that are generally located in only metropolitan areas. In addition, a small number of pharmacists felt not having access to clinicians, or the cost of updating their knowledge or of attending education sessions as barriers to their learning.

## 4. Discussion

The aims of this survey were to understand the level of confidence that pharmacists have in managing patients with IBD, while also identifying any perceived barriers to their exposure to IBD care or to management-related opportunities for further learning. This is the first survey of pharmacists’ confidence about IBD management and perceived barriers to IBD knowledge in Australia. It provides an initial step towards our current understanding of the role of pharmacists and a platform for further research in this area. Our survey highlights the fact that pharmacists are not confident about managing IBD within the current practice model, where their core role is dispensing medicines. Findings suggest that pharmacists’ base level of understanding of IBD is limited and a more comprehensive education is required. While confidence levels can indeed be improved through educational sessions, potential barriers still remain to application in IBD management. 

There is a limited amount of literature relating to primary healthcare providers and the management of IBD [22,24,27]. The available management guidelines may be a helpful resource but are not designed for a primary care setting [28]. A recent finding from a survey reported the importance of understanding barriers and facilitators to patient involvement in IBD research and the significance of their involvement in exploring new treatment opportunities [29,30]. A similar approach could be implemented to investigating perception of healthcare professionals in IBD. The findings from published literature [5,7,14] and this survey, however, show that IBD management, in practice, does not effectively utilise pharmacists as an integral part of a multidisciplinary team, capable of providing much-needed care to patients as demonstrated in other chronic diseases such as diabetes and asthma [31,32,33,34]. Our findings have also provided important insights into pharmacists understanding of and exposure to IBD management, thereby underlining how the role of pharmacists could be optimised. However, adequate knowledge of and clinical experience with this disease is essential for this to be effective in practice.

Individual pharmacists may only provide advice for a few IBD patients on a regular basis, as the majority of IBD care in Australia is currently delivered by specialists [25,28]. Whilst it is important to have specialist care, it tends to put a disproportionate burden on gastroenterologists, who often have to manage all aspects of the disease [14]. Moreover, we found that overall, pharmacists have limited confidence in key areas of IBD management, namely, addressing patients’ needs, providing additional support for IBD patients and making decisions about patient care. There was a reassuringly positive relationship between the IBD-specific education session and greater confidence among pharmacists with IBD in general. This suggests that such disease-specific education could indeed help to improve pharmacists’ views on IBD management, although whether educational interventions alone can improve and maintain pharmacists’ knowledge of and confidence in managing IBD remains questionable.

A lack of understanding and a lack of access to the available literature have created a certain ambiguity surrounding the roles of pharmacists in the management of IBD. The results of our survey showed pharmacists’ interest in topics associated with IBD, which suggests a willingness to learn more about the disease. Pharmacists were keen to know the science of IBD and to be more aware of drug therapies, treatment options, novel and emerging treatments, guidelines on referral pathways and the stepwise approach to managing IBD. As IBD is a complex disease requiring lifelong management, IBD outcomes continue to improve with time as the development of new therapies evolves [25,35]. New treatment options provide new challenges for pharmacists on optimal communication of information and to seamlessly integrate patients’ needs into the conversation. This is a more complex process, as studies have shown that the views of patients differ markedly from those of healthcare professionals [24,35,36,37]. Pharmacists have to be aware of the health literacy needs of patients, and tailor their communication accordingly. Hence, pharmacists’ knowledge and practice are essential resources to achieve better patient outcomes acting as a conduit in the community and could become integral to managing IBD in primary care.

In the current model of practice, pharmacists are mainly confined to dispensing roles with limited opportunities to be directly involved in the care of patients with IBD [38]. Whilst this may be the case, interventions that directly lead to clinical improvements can indeed be achieved by extending and broadening the role of pharmacists. Such interventions pertain to encouraging patient compliance/adherence to therapy, early interventions for flares where patients seek help from pharmacists as the initial contact and monitoring of adverse effects associated with immunomodulators and biologics [7,15,16]. Pharmacists in our survey identified time and information as potential barriers to updating their knowledge on IBD. These perceived barriers of access to and awareness of information as well as limited time may indeed be associated with their low levels of confidence when it comes to managing IBD.

However, there were certain limitations to our study. This exploratory survey utilised a cross-sectional design and provided a ‘snapshot’ of pharmacists’ perceptions of confidence; and we assessed if perceptions were altered by an educational intervention with pre-post surveys. The survey was conducted independent to the content and delivery of the educational session. There is a probable participation bias—only those with an interest in IBD and those directly working in providing care to IBD patients. Like any survey design, there is self-reporting bias. While these are important considerations, bias towards involving those interested in IBD can also be considered a strength of the study as it highlights what is currently lacking in pharmacists’ management of IBD. In addition, this was a single site study of pharmacists attending a conference with the goal to increase their professional competence and findings may not be generalizable. The lack of demographical data limit is another limitation. As other programmed educational sessions were scheduled the survey was tightly time limited and therefore too difficult to collect demographic data.

There are no published studies that have primarily explored the perceptions of pharmacists regarding IBD. This is important, and there is clearly a need for further research to address the gap relating to pharmacists’ knowledge, understanding and awareness of IBD, its management and patient needs. Such research could further explore patients’ and other healthcare professionals’ perceptions as to the role of pharmacists in IBD management and in their care.

## 5. Conclusions

The findings of this survey have clinical implications for pharmacists in managing IBD. Importantly, the results suggest that pharmacists perceive themselves as being not confident enough about IBD management. Education should be provided to pharmacists about the various aspects of IBD management focusing on such topics as treatment options, the stepwise approach, referral pathways, therapeutic decision-making, lifestyle management options, adherence to therapy and patients’ overall wellbeing and health. Continued education could be in form of a webinar or online modules that are readily available and accessible to pharmacists in any location. Despite its obvious limitations, this study has nevertheless provided insight into pharmacists’ understanding of and confidence in the management of patients with IBD. This is particularly useful as, up until now, very little data on the primary care management of IBD has been available. However, more research is needed to explore opportunities for interventions and proactive strategies, by means of which pharmacists’ knowledge and practice could be enhanced and optimized.

## Figures and Tables

**Table 1:** Additional questions of the post-session section of the IBD Confidence Survey.

Description	Questions
Free-text questions	What would you like to learn more about IBD?
	What would be barriers to your learning?
Non-free text question	How likely are you to attend a similar education session on IBD in the future?

**Table 2:** Corresponding statements of the pre- and post-session section the IBD Confidence Survey.

Confidence Statements	
Pre-session statement 1	I am confident with my understanding of IBD and its management.
Post-session 1a	Identifying signs and symptoms of inflammatory bowel disease (IBD).
1b	Distinguishing IBD from other gastrointestinal diseases.
1c	Recognising when referral is required.
Pre-session statement 2	I am able to provide relevant information about IBD and its management.
Post-session 2	Providing advice to patients on the management of IBD.
Pre-session statement 3	I am confident in making decisions with IBD patients about their care.
Post-session 3	Reviewing/monitoring current treatments and provide guidance and tailored information on adherence and compliance.
Pre-session statement 4	I am able to provide additional support in managing patients with IBD.
Post-session 4	Accessing relevant resources on IBD in primary care.
Pre-session statement 5	I feel confident in addressing the needs of IBD patient’s regarding their condition and treatment.
Post-session 5	Addressing the needs of IBD patients regarding their condition and treatment.

## Supplementary Materials

The following are available online at https://www.mdpi.com/2226-4787/8/2/68/s1, Supplementary Information 1. The IBD Confidence Survey.

## Abbreviations

IBD	Inflammatory bowel disease
HCP	healthcare professionals
MOA	mode of action
CM	complementary medicines
