# Supplementary material for: Pharmacists’ Confidence in Managing Patients with Inflammatory Bowel Disease
Source: Pharmacy (Basel). 2020 Apr 17;8(2):68. doi: 10.3390/pharmacy8020068 (PMC7355482; doi:10.3390/pharmacy8020068)
Supplement: Supplementary file 1 [file pharmacy-08-00068-s001.pdf]

Supplementary Information 1:

IBD Confidence Survey

Please complete this section BEFORE the education session on Inflammatory Bowel Disease.

Pre-session Questions:

Please answer the following questions on a scale of 1 to 5. (1 = not confident; 5 = entirely confident).

1. I am confident with my understanding of IBD and its management.

   1 2 3 4 5

2. I am able to provide relevant information about IBD and its management.

   1 2 3 4 5

3. I am confident in making decisions with IBD patients about their care.

   1 2 3 4 5

4. I am able to provide additional support in managing patients with IBD.

   1 2 3 4 5

5. I feel confident in addressing the needs of IBD patient's regarding their condition and treatment.

   1 2 3 4 5

**Disclaimer: This is an independent survey as part of a research project at The University of Newcastle and is not sponsored or endorsed by AbbVie or PSA.**

Please complete this section AFTER the education session on Inflammatory Bowel Disease

Post-session Questions:

Please answer the following questions on a scale of 1 to 5. (1 = not confident; 5 = entirely confident).

1. Identifying signs and symptoms of inflammatory bowel disease (IBD).

   1 2 3 4 5

2. Distinguishing IBD from other gastrointestinal diseases.

   1 2 3 4 5

3. Providing advice to patients on the management of IBD.

   1 2 3 4 5

4. Recognising when referral is required.

   1 2 3 4 5

5. Reviewing/monitoring current treatments and provide guidance and tailored information on adherence and compliance.

   1 2 3 4 5

6. Addressing the needs of IBD patients regarding their condition and treatment.

   1 2 3 4 5

7. Accessing relevant resources on IBD in primary care.

   1 2 3 4 5

8. What would you like to learn more about IBD?

9. What would be barriers to your learning?

10. How likely are you to attend a similar education session on IBD in the future?

    Very unlikely 1 2 3 4 5 Very likely
